# Case report of vaccine-induced anaphylactic reaction reclassified after vaccine challenge

**DOI:** 10.1016/j.jacig.2025.100484

**Published:** 2025-04-22

**Authors:** Maria Aman, Chirine A. Abou Turk, John C. Carlson

**Affiliations:** Ochsner Health System, New Orleans, La

**Keywords:** Vaccine reaction, anaphylaxis, breath holding, vaccine challenge

## Abstract

At age 4 months, an infant had skin changes and cessation of breathing responsive to epinephrine after vaccine administration. The atypical breathing recurred during vaccine challenge but was consistent with breath-holding spell rather than anaphylaxis. Vaccine challenges can be performed in appropriate settings even when cases are consistent with anaphylaxis.

Vaccines have the potential to trigger anaphylaxis, although reactions are exceptionally rare, occurring in approximately 1.31 per million vaccine doses.^1^ The criteria for diagnosis of anaphylaxis after immunization were derived by expert consensus to help in differentiating vaccine reactions from other causes^2^ (Table I^3^^,^^4^). Of those children suspected of having IgE-mediated vaccine reactions, 92% tolerate revaccination, suggesting that most cases of suspected vaccine allergy are due to other conditions.^5^ Conditions that may be mistaken for vaccine-induced anaphylaxis include those that are induced by the psychological stress of vaccination. The term *immunization stress–related response* is used for this category of adverse events, and it includes acute stress reactions, vasovagal reactions, and dissociative neurologic symptom reactions (previously referred to as conversion disorders).^6^

**Table I:** Comparison of signs and symptoms of conditions that may mimic anaphylaxis in infants

Condition	System				
	Skin/mucosa	Respiratory	Cardiovascular	Gastrointestinal	Central nervous system
Anaphylaxis^2^	Urticaria Angioedema of skin Erythema with or without itch Red and/or itchy eyes Cyanosis	Expiratory wheeze Inspiratory stridor Angioedema of the upper airway mucosa Respiratory distress	Hypotension Loss of consciousness	Vomiting Diarrhea	Impairment due to hypotension
Breath holding spells^3^	Cyanosis Pallor Both	Noiseless pause after expiration Labored inspiration	Loss of consciousness		Limpness Opisthotonic posture Body jerks with urinary incontinence
Hypotonic hyporesponsive episode^4^	Pallor Cyanosis		Hyporesponsiveness		Hypotonia

At age 4 months, an otherwise healthy female child received her routine immunizations at her pediatric primary care clinic. The vaccines administered were a combined diphtheria and tetanus toxoid and acellular pertussis (DTaP), hepatitis B, and inactivated polio vaccine (PEDIARIX [GlaxoSmithKline, Philadelphia, Pa]), *Haemophilus* influenzae type B conjugate vaccine, and 20-valent pneumococcal conjugate vaccine, all of which were administered intramuscularly, and an orally administered rotavirus pentavalent vaccine. Approximately 5 minutes after vaccine administration, the patient developed a red, papular rash on her face. She was given cetirizine, 1.25 mg, in the office. After 10 minutes, she developed an unusual breathing pattern and then stopped breathing. She was given epinephrine, 0.15 mg, administered intramuscularly via autoinjector, and an ambulance was called. The duration of apnea was not documented, but it ended after epinephrine administration and without other intervention. The patient was given diphenhydramine and transferred via ambulance to the emergency department. On arrival, the patient was asymptomatic other than having mild tachycardia; her rash had resolved. She was given 1 dose of dexamethasone, 0.6 mg/kg orally, and prescribed a course of famotidine and diphenhydramine for 3 days. She was also provided with an epinephrine intramuscular autoinjector in case of further severe allergic reactions. There was no blood draw to measure her tryptase level.

After 3.5 hours of observation, the patient was discharged directly to the high-risk allergy clinic to be seen the same day. Although reactions to any vaccine are rare, there is a case series of children aged 4 to 17 years reacting to the DTaP vaccine^7^; therefore, it was considered the most likely of the vaccines to have caused an anaphylactic reaction in this patient. After joint decision making, it was determined that our patient’s future 6-month routine vaccines would be carried out in the high-risk allergy clinic using all immunizations except the PEDIARIX vaccine, which contains DTaP and would therefore be administered on a subsequent date. Given the low probability of a reaction and uncertain predictive values, skin testing was not performed.

At age 6 months, the patient was given the Rotavirus pentavalent vaccine, the pneumococcal conjugate 20-valent vaccine, and the *Haemophilus* influenzae type B conjugate vaccine in the high-risk allergy clinic. After administration of the vaccines, she immediately started to cry and had a redness on her face consistent with crying. As her crying subsided, she displayed sleepiness consistent with postcrying tiredness. She then developed an unusual respiration pattern consisting of sharp inhalations through the nose that were spaced 5 to 8 seconds apart, with no sound or visible movement of the chest between inspirations. This pattern lasted for about 3 minutes while her physical examination findings remained otherwise unchanged. Normal respirations resumed as she fell asleep. She awoke in a normal state 30 minutes after immunization. We believed that this was a response to the psychological stress from the pain of immunization rather than an immune response to the vaccines administered.

The patient was given a combined DTaP vaccine, hepatitis B vaccine, and combined inactivated polio vaccine (PEDIARIX) administered intramuscularly in a separate visit to the high-risk allergy clinic because the latter was the vaccine most likely to trigger an anaphylactic reaction. The patient tolerated administration of this vaccine when it was given in conjunction with bottle feeding. At this stage, the patient was cleared to receive future vaccines at her pediatrician’s office, with the recommendation that she have bottles or other distractions for future vaccinations to lessen the chance of similar reactions.

Initially, the patient appeared to meet the diagnostic criteria for anaphylaxis owing to cessation of breath and skin findings, although the criteria for respiratory involvement would technically require wheeze, stridor, airway angioedema, or 2 alternate signs of respiratory distress.^2^ No measurement of acute tryptase level was available to help confirm the diagnosis. Rechallenge resulted in a cessation of breathing very similar to the initial reaction but without apparent skin changes. Because sudden cessation of breathing without other signs is not a manifestation of anaphylaxis, we did not treat the reaction, which resolved after sleep. We made a presumptive diagnosis of a breath-holding spell (BHS).

BHSs are a common phenomenon among children, typically occurring in those younger than 12 months, and they usually resolve by age 8 years.^3^ BHS events are involuntary, with mild cases involving noiseless expiration and color change, whereas severe cases involve loss of consciousness. There is typically an inciting event such as pain or anger, sometimes followed by a brief period of vocalized crying, which gives way to noiseless expiration. This may be followed by a color change and deepening cyanotic or pallor (or both) and, in severe cases, a lack of consciousness. This may be followed by jerking of the child’s body and urinary incontinence. The BHS episode ends with resumption of breathing. The diagnosis of BHS does not explain the redness or papular rash that appeared on the patient’s face and resolved with epinephrine during the index event; cyanosis or pallor are more common. However, it was the cessation of breathing that alarmed the family and physician team sufficiently for epinephrine administration, and it was replication of this cessation of breathing with readministration of vaccines that led to this presumptive diagnosis.

BHSs were not included in the description of immunization stress–related response.^6^ However, they have been postulated to be a cause of adverse reactions with immunization.^4^^,^^8^ Among children who have BHSs, the first spell nearly always occurs before age 2 years, whereas approximately 5% of breath holders have their first spell before age 2 months.^3^ Hypotonic hyporesponsiveness episodes are a more commonly reported adverse event after vaccination, particularly with whole cell pertussis-containing immunizations.^4^ These episodes do not include the changes in respiration demonstrated by our patient.

We were comfortable with readministration of vaccines, in part because our system has a model for consolidating high-risk allergy procedures in our hospital-based clinics.^9^ This case highlights the value of rechallenge with vaccines even in cases in which there is a high suspicion of anaphylaxis. Given the low probability of IgE-mediated reactions with vaccines and the relatively high prevalence of stress responses to immunization, consideration should be given to rechallenge, even in patients who may meet the criteria for anaphylaxis. The family consented to publication of this case report to raise awareness of this phenomenon.

## Disclosure statement

Disclosure of potential conflict of interest: The authors declare that they have no relevant conflicts of interest.
